# The causal relationship between gastro-oesophageal reflux disease and idiopathic pulmonary fibrosis: a bidirectional two-sample Mendelian randomisation study

**DOI:** 10.1183/13993003.01585-2022

**Published:** 2023-05-25

**Authors:** Carl J. Reynolds, Fabiola Del Greco M, Richard J. Allen, Carlos Flores, R. Gisli Jenkins, Toby M. Maher, Philip L. Molyneaux, Imre Noth, Justin M. Oldham, Louise V. Wain, Jiyuan An, Jue-Sheng Ong, Stuart MacGregor, Tom A. Yates, Paul Cullinan, Cosetta Minelli

**Affiliations:** 1 National Heart and Lung Institute, Imperial College London, London, UK; 2 Institute for Biomedicine, Eurac Research, Bolzano, Italy; 3 Department of Population Health Sciences, University of Leicester, Leicester, UK; 4 National Institute for Health Research, Leicester Respiratory Biomedical Research Centre, Glenfield Hospital, Leicester, UK; 5 Research Unit, Hospital Universitario Nuestra Señora de Candelaria, Santa Cruz de Tenerife, Spain; 6 CIBER de Enfermedades Respiratorias, Instituto de Salud Carlos III, Madrid, Spain; 7 Genomics Division, Instituto Tecnológico y de Energías Renovables, Santa Cruz de Tenerife, Spain; 8 Faculty of Health Sciences, University of Fernando Pessoa Canarias, Las Palmas de Gran Canaria, Spain; 9 Keck School of Medicine, University of Southern California, Los Angeles, CA, USA; 10 Division of Pulmonary and Critical Care Medicine, University of Virginia, Charlottesville, VA, USA; 11 Department of Internal Medicine, Division of Pulmonary and Critical Care Medicine, University of Michigan, Ann Arbor, MI, USA; 12 Centre for Agriculture and the Bioeconomy, Faculty of Science, Queensland University of Technology, Brisbane, Australia; 13 Population Health Department, QIMR Berghofer Medical Research Institute, Herston, Australia; 14 Division of Infection and Immunity, Faculty of Medicine, University College London, London, UK

## Abstract

**Background:**

Gastro-oesophageal reflux disease (GORD) is associated with idiopathic pulmonary fibrosis (IPF) in observational studies. It is not known if this association arises because GORD causes IPF or because IPF causes GORD, or because of confounding by factors, such as smoking, associated with both GORD and IPF. We used bidirectional Mendelian randomisation (MR), where genetic variants are used as instrumental variables to address issues of confounding and reverse causation, to examine how, if at all, GORD and IPF are causally related.

**Methods:**

A bidirectional two-sample MR was performed to estimate the causal effect of GORD on IPF risk and of IPF on GORD risk, using genetic data from the largest GORD (78 707 cases and 288 734 controls) and IPF (4125 cases and 20 464 controls) genome-wide association meta-analyses currently available.

**Results:**

GORD increased the risk of IPF, with an OR of 1.6 (95% CI 1.04–2.49; p=0.032). There was no evidence of a causal effect of IPF on the risk of GORD, with an OR of 0.999 (95% CI 0.997–1.000; p=0.245).

**Conclusions:**

We found that GORD increases the risk of IPF, but found no evidence that IPF increases the risk of GORD. GORD should be considered in future studies of IPF risk and interest in it as a potential therapeutic target should be renewed. The mechanisms underlying the effect of GORD on IPF should also be investigated.

## Abstract 2

**This bidirectional two-sample Mendelian randomisation study provides strong evidence that gastro-oesophageal reflux disease (GORD) increases the risk of idiopathic pulmonary fibrosis (IPF), but found no evidence that IPF increases the risk of GORD** https://bit.ly/3Lde737

## Introduction

Idiopathic pulmonary fibrosis (IPF) is a progressive fibrotic lung disease characterised by a usual interstitial pneumonia pattern on thorax computed tomography or biopsy in the absence of a recognised cause [1]. The incidence in Europe and North America is 3–9 per 100 000 person-years [2] and the prognosis is poor, with a median survival of 3 years [3, 4]. IPF is thought to result from epithelial injury in the distal airways, in a susceptible host, initiating a dysregulated repair process [5]. Environmental contributions to IPF are not well understood but several exposures have been posited as causal, including smoking, diabetes mellitus and gastro-oesophageal reflux disease (GORD).

GORD is defined by nonphysiological aspiration of gut contents associated with troublesome symptoms and/or complications such as oesophagitis [6]. GORD has been associated with IPF in observational studies [7], but it is not known if GORD causes an increased risk of IPF. The observed association might be due to confounding by factors, such as smoking, associated with both GORD and IPF. The association could also result from reverse causation, with IPF causing an increased risk of GORD rather than *vice versa* [8]. This is plausible given that reduced lung compliance in IPF can lead to more negative intrathoracic pressures and reflux events are inversely correlated with inspiratory thoracic pressures [9, 10].

Unlike observational associations, genetic associations are not affected by classical confounding or reverse causation, as genes are randomly allocated at conception. A Mendelian randomisation (MR) approach that uses genetic variants known to affect GORD as its proxies (instrumental variables) can therefore provide indirect evidence for a causal effect of GORD on the risk of IPF, if its underlying assumptions hold [11, 12]. Likewise, using genetic variants known to affect IPF risk as its proxies, the same approach can provide indirect evidence for a causal effect of IPF on the risk of GORD.

Here we describe a bidirectional, two-sample MR study to estimate the causal effect of GORD on the risk of IPF and of IPF on the risk of GORD. We used data from the largest available genome-wide association meta-analyses for both GORD [13] and IPF [14].

## Methods

### Genetic data

**FIGURE 1:**
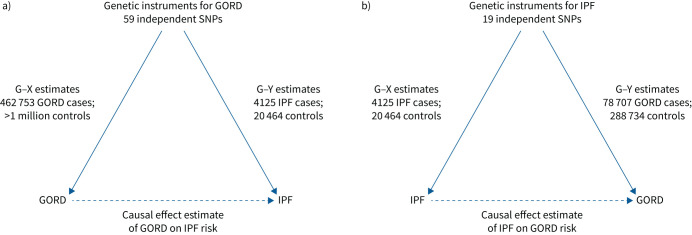
Overview of our two-sample Mendelian randomisation analysis of a) gastro-oesophageal reflux disease (GORD) on idiopathic pulmonary fibrosis (IPF) risk and b) IPF on GORD risk. SNP: single nucleotide polymorphism; G–X: gene–exposure association; G–Y: gene–outcome association.

For both our analyses of the effect of GORD on IPF risk and of the effect of IPF on GORD risk we used two-sample MR where summary statistics (effect estimates and standard errors) for the gene–exposure (G–X) and gene–outcome (G–Y) associations were obtained from separate studies. A graphical overview of the two MR analyses is provided in figure 1.

For the MR of the effect of GORD on IPF risk, instruments were selected from the largest available genome-wide association study (GWAS) meta-analysis on GORD by Ong *et al.* [13]. For each instrument (single nucleotide polymorphism (SNP)), summary statistics of the G–X association (expressed as log odds ratio for GORD) were obtained from the replication stage of Ong *et al.* [13]. Summary statistics of the G–Y association (log odds ratio for IPF) were obtained from the authors of the GWAS meta-analysis on IPF [14].

Similarly, for the MR of the effect of IPF on GORD risk, instruments were selected from the largest available GWAS meta-analysis on IPF by Allen *et al*. [14]. For each SNP, summary statistics of the G–X association (log odds ratio for IPF) were obtained from this GWAS, while summary statistics of the G–Y association (log odds ratio for GORD) were obtained from the authors of the GWAS meta-analysis on GORD [13].

### MR methods

Here we provide an overview of the MR methods used, with a more detailed description of these methods, their underlying assumptions, relevant references and the code used for the analyses reported in the supplementary material.

We estimated the causal effect of GORD on IPF risk (figure 1a) and of IPF on GORD risk (figure 1b) by first deriving SNP-specific MR estimates using the Wald estimator (G–Y divided by G–X) and then pooling them using inverse variance weighted, fixed effect meta-analysis (IVW-FE).

We used the IVW-FE method for our main MR analyses as this is the most powerful method, but it assumes absence of pleiotropy, *i.e.* variants chosen as instruments for the exposure cannot affect the outcome through any other independent pathways. Pleiotropy can bias MR findings and we therefore investigated its possible presence through assessment of the heterogeneity in the MR estimates across SNPs, using the I^2^-index and Cochran's Q heterogeneity test.

In the presence of pleiotropy, possible pleiotropic SNPs were identified graphically based on their contribution to the overall heterogeneity (Cochran's Q-statistic) and we repeated the IVW-FE analysis after removing the pleiotropic SNPs. We also performed MR analyses on all SNPs using methods that can account for pleiotropy under different assumptions about its nature. In particular, we considered the following methods: inverse variance weighted random effect, weighted median, weighted mode-based and MR-Egger, with the simulation extrapolation (SIMEX) method to correct for measurement error (dilution bias) used if needed.

To provide additional assurance regarding pleiotropy we used PhenoScanner (www.phenoscanner.medschl.cam.ac.uk) to check if instruments used were associated with potential confounders for the effect of GORD on IPF and of IPF on GORD. We performed a leave-one-out analysis to check if any individual SNP was driving the observed association for both GORD on IPF and IPF on GORD.

## Results

Demographic data for the cohorts used is provided in table 1 and described in the following subsections.

**TABLE 1:** Summary information for the studies contributing to the data used in both Mendelian randomisation analyses

**Study**	**Participants (n)**	**Age (years)**	**Male (%)**	**Ever-smoker (%)**	**Genotyping array**	**Imputation panel**
**GORD**						
UK						
Cases	75 720	59			Affymetrix UK BiLEVE array	HRC
Controls	278 565	57	46	41	Affymetrix UK BiLEVE array	
Australia						
Cases	2987	56.5			Illumina Global Screening Array	HRC
Controls	10 169	56	46	54	Illumina Global Screening Array	
**IPF**						
Chicago						
Cases	541	68±3	71	72	Affymetrix SNP Array 6.0	HRC
Controls	542	63±7.5	47	42	Affymetrix SNP Array 6.0	
Colorado						
Cases	1515	66±9.5	68		Illumina Human 660W Quad BeadChip	HRC
Controls	4683		49		Illumina Human 660W Quad BeadChip	
UK						
Cases	612	70±8.4	71	73	Affymetrix UK BiLEVE array	HRC
Controls	3366	65±5.5	70	70	Affymetrix UK BiLEVE and UK Biobank arrays	
UUS						
Cases	793	69±8.1	75	69	Affymetrix UK Biobank and Spain Biobank arrays	HRC
Controls	9999	58±7.8	72	68	Affymetrix UK BiLEVE and UK Biobank arrays	
Genentech						
Cases	664	68±7.5	74	67	Illumina HiSeq X Ten platform	
Controls	1874	56±9.3	27	18	Illumina HiSeq X Ten platform	

Data for age are presented as mean±sd. GORD: gastro-oesophageal reflux disease; IPF: idiopathic pulmonary fibrosis; UUS: USA, UK and Spain; HRC: Haplotype Reference Consortium.

### Effect of GORD on IPF risk

The GWAS on GORD by Ong *et al*. [13] identified 59 independent genome-wide significant (p<5×10^−8^) SNPs, based on a total sample of 78 707 GORD cases and 288 734 controls, with replication of findings in 462 753 cases and 1 484 025 controls [13]. The sample was almost entirely of White European ancestry. GORD cases were defined by having one or more of a GORD International Classification of Diseases, 10th Revision code, GORD self-report or use of GORD medication.

Of the 59 SNPs, two were missing in the IPF GWAS meta-analysis dataset and a proxy (*i.e.* SNP with linkage disequilibrium (LD) r^2^≥0.8 with the SNP of interest) was used instead (supplementary table S1). All SNPs used in the MR analysis were “strong” instruments with an F-statistic >10 [15], where the F-statistic is a function of both magnitude and precision of the SNP's effect on GORD [16]. Individual F-statistics ranged from 12 to 81 (supplementary table S1). PhenoScanner results for the SNPs used showed that none were associated with potential confounders for the effect of GORD on IPF (in bold in supplementary table S3). However, none of these possibly pleiotropic instruments had an impact on the results, as demonstrated by a negative leave-one-out analysis (no influential instruments).

This MR analysis showed that GORD increases the risk of IPF, with an OR of 1.61 (95% CI 1.04–2.49; p=0.032).

There was no statistical evidence of pleiotropy, with an I^2^ of 0% (95% CI 0–31%) and a heterogeneity p-value of 0.52.

### Effect of IPF on GORD risk

The GWAS meta-analysis on IPF by Allen *et al*. [14] included three previously published studies (Chicago, Colorado and UK) [17–19] plus an unpublished study including independent case–control studies from the USA, UK and Spain, and a study of clinical trial subjects, the Genentech study. The Genentech study consisted of cases from three IPF clinical trials and controls from four non-IPF clinical trials.

The GWAS meta-analysis included a total of 4125 IPF cases and 20 464 controls of White European ancestry. IPF cases were defined using the joint American Thoracic Society/European Respiratory Society guidelines [1, 20, 21].

Of the 19 SNPs, 15 were missing in the GORD GWAS meta-analysis database. For nine a proxy (LD r^2^>0.8) was used (supplementary table S2). Data for the remaining six, for which a satisfactory proxy could not be identified, were obtained from the authors. Individual F-statistics ranged from 30 to 1721 (supplementary table S2). PhenoScanner results for the SNPs used showed that none were associated with potential confounders for the effect of IPF on GORD (supplementary table S4).

The MR analysis showed no evidence of a causal effect of IPF on the risk of GORD, with an OR of 0.999 (95% CI 0.997–1.001; p=0.245).

**FIGURE 2:**
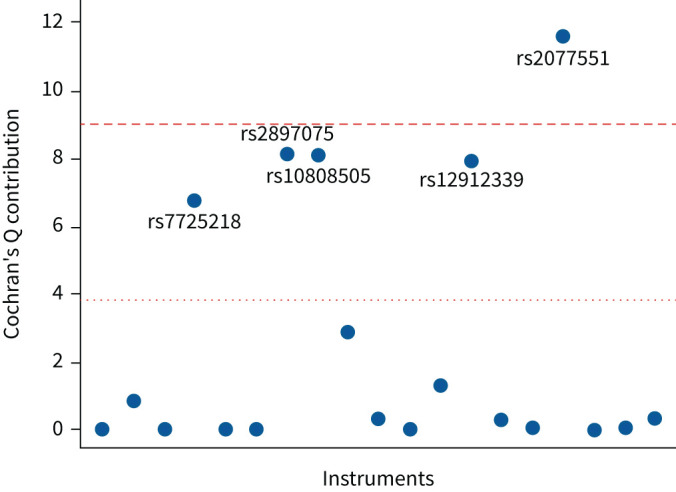
Identification of pleiotropic instruments in the Mendelian randomisation on the effect of idiopathic pulmonary fibrosis on gastro-oesophageal reflux disease. Individual variant contributions to Cochran's Q heterogeneity statistic with the 5th (dotted line) and Bonferroni corrected (0.05/19th) percentiles (dashed line) of a Chi-squared with 1 degree of freedom.

**FIGURE 3:**
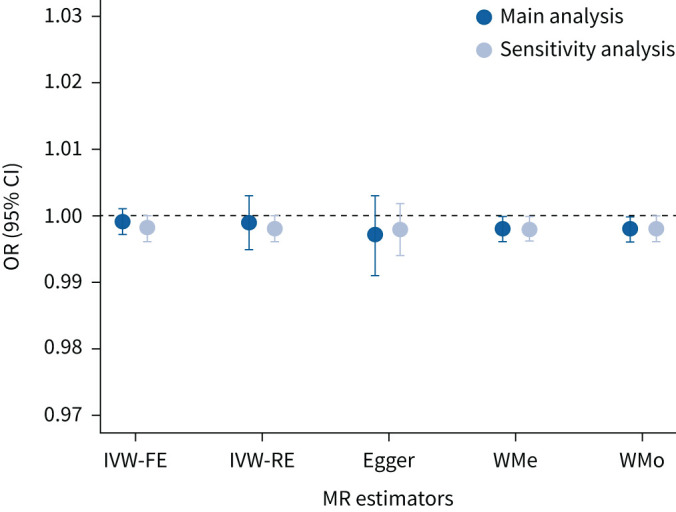
Results of the Mendelian randomisation (MR) on the effect of idiopathic pulmonary fibrosis on gastro-oesophageal reflux disease risk using different robust methods to address the issue of pleiotropy: main analysis and sensitivity analysis with five possible pleiotropic single nucleotide polymorphisms removed. IVW-FE: inverse variance weighted fixed effect; IVW-RE: inverse variance weighted random effect; WMe: weighted median; Egger: MR-Egger; WMo: weighted mode-based.

We found evidence of pleiotropy, with an I^2^ of 63% (95% CI 40–77%) and a heterogeneity p-value of 0.00016. We identified five possible pleiotropic SNPs, shown in figure 2. After removing the SNPs, there was no evidence of residual pleiotropy (I^2^=0%; heterogeneity p=0.944) and the results remained null (OR 0.998, 95% CI 0.996–1.001; p=0.137). Similar null findings were obtained when using robust methods that adjust for pleiotropy, as shown in figure 3.

## Discussion

We undertook a bidirectional two-sample MR study to investigate the causal relationship between GORD and IPF. We found evidence of a causal effect of GORD on IPF, but not of IPF on GORD.

The odds of IPF were 1.6 times higher in the presence of GORD, although there was substantial uncertainty around the estimate (95% CI 1.04–2.49) and, in general, the magnitude of the causal effect obtained from MR studies should be interpreted with caution [22]. Moreover, while lifetime risk of IPF is increased by exposure to genetically determined GORD, this cannot be used to directly infer the possible effect on IPF risk of an intervention on GORD. Previous evidence for the association of GORD and IPF arises from observational studies where it has not been possible to confidently make causal inferences because of the potential for residual confounding and reverse causation. A case–control study of 17 consecutive biopsy-proven IPF patients and eight controls with non-IPF interstitial lung disease (ILD) at a US tertiary ILD centre [23], which involved ambulatory oesophageal pH monitoring and a GORD symptom questionnaire, found that the majority of IPF, but not non-IPF ILD, patients had abnormal oesophageal acid exposure which was usually clinically silent. A similar pattern was seen in a later study of 65 consecutive IPF patients at the same centre, which used 133 consecutive asthma patients referred to a gastrointestinal motility clinic as controls [24]. A systematic review of IPF comorbidities found 23 studies that reported prevalence of GORD in IPF patients. The majority of the studies were small, with fewer than 50 patients, and they used a variety of case definitions for GORD [25]. Study estimates varied widely, from 0% to 94%, but most were around 30% [25]. This is higher than prevalence estimates for the general population; GORD global population prevalence increases with age and peaks at 18% for people aged 75–79 years [26]. A recent meta-analysis of 18 case–control studies [7] comprising 3206 patients with IPF and 9368 controls, found the odds of IPF was 2.94 times higher in people with GORD (95% CI 1.95–4.42), but with high heterogeneity across studies (I^2^=86%; heterogeneity p<0.00001). The authors reported that confounding by smoking was likely for two reasons. First, a post-hoc meta-regression that controlled for smoking found the association was no longer significant (OR 0.66, 95% CI 0.34–1.27). Second, the effect sizes tended to be larger in studies where a higher proportion of cases, and a lower proportion of controls, smoked. There was significant correlation between the ratio of the proportion of smokers (and ex-smokers) in IPF cases over the proportion in controls and the log odds ratio for the association of GORD and IPF. Our findings using an MR approach overcome confounding by smoking and any other (known or unknown) confounding of observational analyses to indicate a causal effect of GORD on IPF risk. As genetic predisposition to GORD is present from birth, MR estimates the effect of an individual's lifelong predisposition to GORD on the risk of IPF and our findings are therefore also independent of any current nutritional or pharmacological treatment for GORD.

The underlying mechanism explaining how GORD may increase IPF risk is unknown; however, aspiration of gastric contents can cause chemical pneumonitis or aspiration pneumonia [27]. One study of GORD in IPF included bronchoalveolar lavage (BAL) and compared 21 IPF patients with 20 non-IPF ILD patients and 16 patients undergoing bronchoscopy for other diseases. BAL fluid was analysed for the presence of pepsin and bile acids. Pepsin and/or bile acids were present in 62% of IPF patients compared with 25% of non-IPF ILD patients and were absent for all patients undergoing bronchoscopy for other diseases [28].

While direct chemical or bacterial epithelial insult may initiate or promote fibrosis, another possibility is by indirect means. For example, GORD may increase IPF risk through airway acidification secondary to aspiration disrupting mucin 5B (MUC5B) function and impairing innate immunity. The *MUC5B* promoter variant rs35705950, which increases airway expression of MUC5B, is the strongest common identified risk factor for IPF. The odds of developing pulmonary fibrosis are 5 times higher for individuals carrying one copy of the disease allele, rising to 20 times higher for individuals with two copies, when compared with individuals carrying no copies of the disease allele. The frequency of the disease allele is around 9% in European ancestry populations [29, 30]. While MUC5B is undoubtedly important for IPF risk, the mechanisms by which increased airway MUC5B expression increases IPF risk are not well understood. In evolutionary terms, it is a highly conserved airway mucin that plays a key role in antimicrobial host defence [31], and has been shown to reduce mucosal bacterial load and to promote mucosal microbial diversity [32]. The structure, and function, of MUC5B is pH dependent [33, 34] and may be disrupted by acidification of the airway secondary to GORD. In IPF, there are higher airway bacterial loads and reduced microbial diversity compared with COPD patients and healthy controls [35], and this is associated with disease progression [36].

The theoretical possibility of reverse causation, whereby the association between IPF and GORD is driven by IPF, rather than GORD, is well described [37]. Restrictive lung disease may distort the oesophageal gastric junction and predispose to hiatus hernia. Indeed, hiatus hernia has been observed to be more common in IPF than in patients with asthma or COPD [38]. Reduced lung compliance in IPF can result in increasingly negative intrathoracic pressures being required for inspiration and an increased gastro-oesophageal pressure gradient [39]. Reflux events generally occur during inspiration and are inversely correlated with inspiratory thoracic pressures [9, 10]. Other mechanical factors that might contribute to GORD secondary to IPF are reduced oesophageal body motility, lower basal lower oesophageal sphincter pressure and delayed gastric emptying [37]. We therefore investigated the possibility of causality in both directions using a bidirectional MR approach, but we found no evidence of a causal effect of IPF on GORD risk. In this MR analysis of the effect of IPF on GORD, however, pleiotropy was observed and five possible pleiotropic SNPs identified, but the result remained null after removing them and when adjusting for pleiotropy using robust methods.

A major strength of our MR work, in contrast to previous observational work examining GORD–IPF relations, is that MR is not affected by classical confounders, such as smoking, or by reverse causation. Our work therefore overcomes these limitations of observational studies to establish GORD as a risk factor for IPF.

Our study also has some limitations. One major problem of MR is that while it is not vulnerable to classical confounding or reverse causation, it is affected by pleiotropy. We found evidence for pleiotropy in our MR of IPF on GORD but adjusting for it did not alter our results. Our study had more power for finding an effect of GORD on IPF than IPF on GORD as a consequence of GORD being more common, and there being many more genotyped GORD patients than IPF patients available. Nevertheless, our confidence interval for the GORD on IPF effect is wide and a future study when larger GWAS data are available would be helpful. We cannot exclude that a small effect of IPF on GORD might be found in a future MR study performed on data from a larger IPF sample.

In interpreting the finding of a causal effect of GORD on IPF, we need to consider the potential impact of the overlap of participants in the control group, as UK Biobank contributed controls to both the GORD and the IPF GWAS meta-analyses. With participant overlap in a two-sample MR, any weak instrument bias would bias the results away from the null, unlike with completely independent samples where the bias is towards the null [40]. However, weak instrument bias is unlikely in our MR (all instruments were above the commonly used F-statistic threshold of >10) and the estimates of the genetic effects on GORD were derived from the GWAS replication stage (no winner's curse), which further reduces the risk of weak instrument bias [41]. Finally, participants contributing to the GWAS meta-analyses used were almost entirely of White European ancestry, limiting generalisability to other groups.

GORD should be taken into account in future studies of IPF risk and interest in it as a potential therapeutic target should be renewed. Treatment of GORD is not well established to be of benefit in IPF; a recent systematic review found no evidence that treating GORD with antacids, or fundoplication, improved outcomes in IPF [42]. There is also potential for harm, since adverse events including increased risk of bacterial infection have been associated with antacid use [42]. Consequently, international guidelines have been updated to include a conditional recommendation against, rather than for, antacid treatment in IPF [43]. The guidelines also highlighted the need for further research to address the risk of bias arising from confounders in observational studies linking GORD and IPF, which we have addressed here by using an MR approach. Ultimately, uncertainty still remains regarding treatment benefit due to a lack of adequately powered well-designed randomised controlled trials (RCTs) in this area, although one is now underway (ClinicalTrials.gov: NCT04965298).

We have assessed the risk of developing IPF in GORD, which is not amenable to indirect assessment by an RCT of GORD treatment, given how rare IPF is an outcome. It could be that GORD has also an independent effect on IPF disease progression; this could be investigated in a future study when sufficient genetic data become available. Understanding the mechanism for increased IPF risk in GORD is now necessary to understand which patients may benefit from existing treatments and for identification of novel therapeutic targets.

### Conclusions

By means of a bidirectional two-sample MR study we have overcome limitations inherent to observational studies and shown that GORD causes an increased risk of IPF, while we found no evidence that IPF causes an increased risk of GORD. GORD should be considered in future studies of IPF risk and interest in it as a potential therapeutic target should be renewed. The mechanisms underlying the effect of GORD on IPF should also be investigated.

## Supplementary material

10.1183/13993003.01585-2022.Supp1 **Please note:** supplementary material is not edited by the Editorial Office, and is uploaded as it has been supplied by the author.
Supplementary tables ERJ-01585-2022.Tables
Supplementary methods ERJ-01585-2022.Methods

## Shareable PDF

10.1183/13993003.01585-2022.Shareable1 This one-page PDF can be shared freely online.
Shareable PDF ERJ-01585-2022.Shareable
