# Supplementary material for: The causal relationship between gastro-oesophageal reflux disease and idiopathic pulmonary fibrosis: a bidirectional two-sample Mendelian randomisation study
Source: Eur Respir J. 2023 May 25;61(5):2201585. doi: 10.1183/13993003.01585-2022 (PMC10209472; doi:10.1183/13993003.01585-2022)
Supplement: Supplementary file 3 [file ERJ-01585-2022.Shareable.pdf]

SHAREABLE PDF

The causal relationship between gastro-oesophageal reflux disease and idiopathic pulmonary fibrosis: a bidirectional two-sample Mendelian randomisation study

Carl J. Reynolds [1], Fabiola Del Greco M [2], Richard J. Allen [3,4], Carlos Flores [5,6,7,8], R. Gisli Jenkins [1], Toby M. Maher [1,9], Philip L. Molyneaux [1], Imre Noth [10], Justin M. Oldham [11], Louise V. Wain [3,4], Jiyuan An [12], Jue-Sheng Ong [13], Stuart MacGregor [13], Tom A. Yates [14], Paul Cullinan [1] and Cosetta Minelli [1]

[1]National Heart and Lung Institute, Imperial College London, London, UK. [2]Institute for Biomedicine, Eurac Research, Bolzano, Italy. [3]Department of Population Health Sciences, University of Leicester, Leicester, UK. [4]National Institute for Health Research, Leicester Respiratory Biomedical Research Centre, Glenfield Hospital, Leicester, UK. [5]Research Unit, Hospital Universitario Nuestra Señora de Candelaria, Santa Cruz de Tenerife, Spain. [6]CIBER de Enfermedades Respiratorias, Instituto de Salud Carlos III, Madrid, Spain. [7]Genomics Division, Instituto Tecnológico y de Energías Renovables, Santa Cruz de Tenerife, Spain. [8]Faculty of Health Sciences, University of Fernando Pessoa Canarias, Las Palmas de Gran Canaria, Spain. [9]Keck School of Medicine, University of Southern California, Los Angeles, CA, USA. [10]Division of Pulmonary and Critical Care Medicine, University of Virginia, Charlottesville, VA, USA. [11]Department of Internal Medicine, Division of Pulmonary and Critical Care Medicine, University of Michigan, Ann Arbor, MI, USA. [12]Centre for Agriculture and the Bioeconomy, Faculty of Science, Queensland University of Technology, Brisbane, Australia. [13]Population Health Department, QIMR Berghofer Medical Research Institute, Herston, Australia. [14]Division of Infection and Immunity, Faculty of Medicine, University College London, London, UK.

Corresponding author: Carl J. Reynolds (carl.reynolds@imperial.ac.uk)

Check for updates

Shareable abstract (@ERSpublications)
This bidirectional two-sample Mendelian randomisation study provides strong evidence that gastro-oesophageal reflux disease (GORD) increases the risk of idiopathic pulmonary fibrosis (IPF), but found no evidence that IPF increases the risk of GORD https://bit.ly/3Lde737

Cite this article as: Reynolds CJ, Del Greco M F, Allen RJ, *et al*. The causal relationship between gastro-oesophageal reflux disease and idiopathic pulmonary fibrosis: a bidirectional two-sample Mendelian randomisation study. *Eur Respir J* 2023; 61: 2201585 [DOI: 10.1183/13993003.01585-2022].

This single-page version can be shared freely online.

Copyright ©The authors 2023.

This version is distributed under the terms of the Creative Commons Attribution Licence 4.0.

This article has an editorial commentary: https://doi.org/10.1183/13993003.00566-2023

Received: 30 Aug 2022
Accepted: 07 March 2023

Abstract

Background Gastro-oesophageal reflux disease (GORD) is associated with idiopathic pulmonary fibrosis (IPF) in observational studies. It is not known if this association arises because GORD causes IPF or because IPF causes GORD, or because of confounding by factors, such as smoking, associated with both GORD and IPF. We used bidirectional Mendelian randomisation (MR), where genetic variants are used as instrumental variables to address issues of confounding and reverse causation, to examine how, if at all, GORD and IPF are causally related.

Methods A bidirectional two-sample MR was performed to estimate the causal effect of GORD on IPF risk and of IPF on GORD risk, using genetic data from the largest GORD (78 707 cases and 288 734 controls) and IPF (4125 cases and 20 464 controls) genome-wide association meta-analyses currently available.

Results GORD increased the risk of IPF, with an OR of 1.6 (95% CI 1.04–2.49; p=0.032). There was no evidence of a causal effect of IPF on the risk of GORD, with an OR of 0.999 (95% CI 0.997–1.000; p=0.245).

Conclusions We found that GORD increases the risk of IPF, but found no evidence that IPF increases the risk of GORD. GORD should be considered in future studies of IPF risk and interest in it as a potential therapeutic target should be renewed. The mechanisms underlying the effect of GORD on IPF should also be investigated.
